# Prune belly syndrome: A rare case report

**DOI:** 10.1002/ccr3.8922

**Published:** 2024-06-17

**Authors:** Siddinath Gyawali, Balkrishna Gyawali, Bhumika Ghimire, Bibek Shrestha, Pratima Khanal, Geha Raj Dahal, Dinesh Prasad Koirala

**Affiliations:** ^1^ Institute of Medicine, Tribhuvan University Teaching Hospital Kathmandu Nepal; ^2^ Manmohan Memorial Institute of Health Sciences Kathmandu Nepal

**Keywords:** congenital disorder, cryptorchidism, omphalocele, prune belly syndrome

## Abstract

**Key Clinical Message:**

In babies presenting with an omphalocele, other components of the prune belly syndrome should be scrutinized for early diagnosis and timely intervention.

**Abstract:**

A male baby on his 13th day of life presented with an omphalocele. On evaluation, he had congenital absence of left kidney and bilateral cryptorchidism. Therefore, he was diagnosed with prune belly syndrome. He responded well to abdminoplasty, and wait and watch policy was applied for his cryptorchidism.

## 1 INTRODUCTION

The term “Prune belly syndrome (PBS)” was coined by Osler in 1895, although in 1839, Frolich first described a case of congenital absence of abdominal wall musculature. Later in 1950, a total of nine similar cases were reported by Eagle and Barrett who named it Eagle and Barrett syndrome. This condition was also called by different other names including triad syndrome and abdominal musculature deficiency syndrome.^1^ It is a rare congenital disorder with an incidence of 3.8 cases per 100,000 live births in males and 1.1 per 100,000 in females.^2^, ^3^ This syndrome consists of a classical triad of urinary tract malformation, bilateral cryptorchidism, and absence of the anterior abdominal wall muscles. However, the majority of affected females do not exhibit identical abdominal wall and urinary tract abnormalities.^1^, ^4^

The pathogenesis of PBS has not been well‐defined yet, though different theories have been described. These theories are based on the developmental urethral obstruction or a mesodermal developmental defect. However, these theories fail to explain the mechanism of occurrence of all the components of the triad and the involvement of other systems in some patients. Therefore, some scientists believe that more than one mechanism, guided by a genetic abnormality, is responsible for causing PBS.^5^, ^6^, ^7^ A study believes that mutation in HNF1β (hepatocyte nuclear factor‐1 beta) gene on chromosome 17q12 might be responsible for causing PBS.^8^

Herein, we report a case of a neonate with protrusion of mass via umbilical region (abdominal wall defect), absent left kidney (urinary tract abnormality), and bilateral cryptorchidism, the classical triad of PBS.

## 2 CASE HISTORY/EXAMINATION

A male baby on his 13th day of life was referred to our center for protrusion of a mass from the umbilical region and abdominal distension since birth. He was born to a 24‐year‐old gravida 3, para 2, and living 2 (G_3_P_2_L_2_) female. The baby was delivered outside our center via normal labor, at term with a birth weight of 2 kg. The mother was on regular antenatal checkups (ANCs) with an unremarkable antenatal history and laboratory results. There was no history of exposure to teratogenic drugs, radiation, or illness with fever and rashes during the pregnancy. Additionally, the mother had no known history of diabetes and there was no history of congenital anomalies in her family. The baby cried immediately after birth and breastfeeding was started immediately. He passed stool and urine within 24 h. Outside our center, the child was treated with intravenous cefotaxime and intravenous fluids. He was then referred to our center for further workup and management of the protruded mass seen on the umbilical region.

## 3 METHODS

**FIGURE 1:**
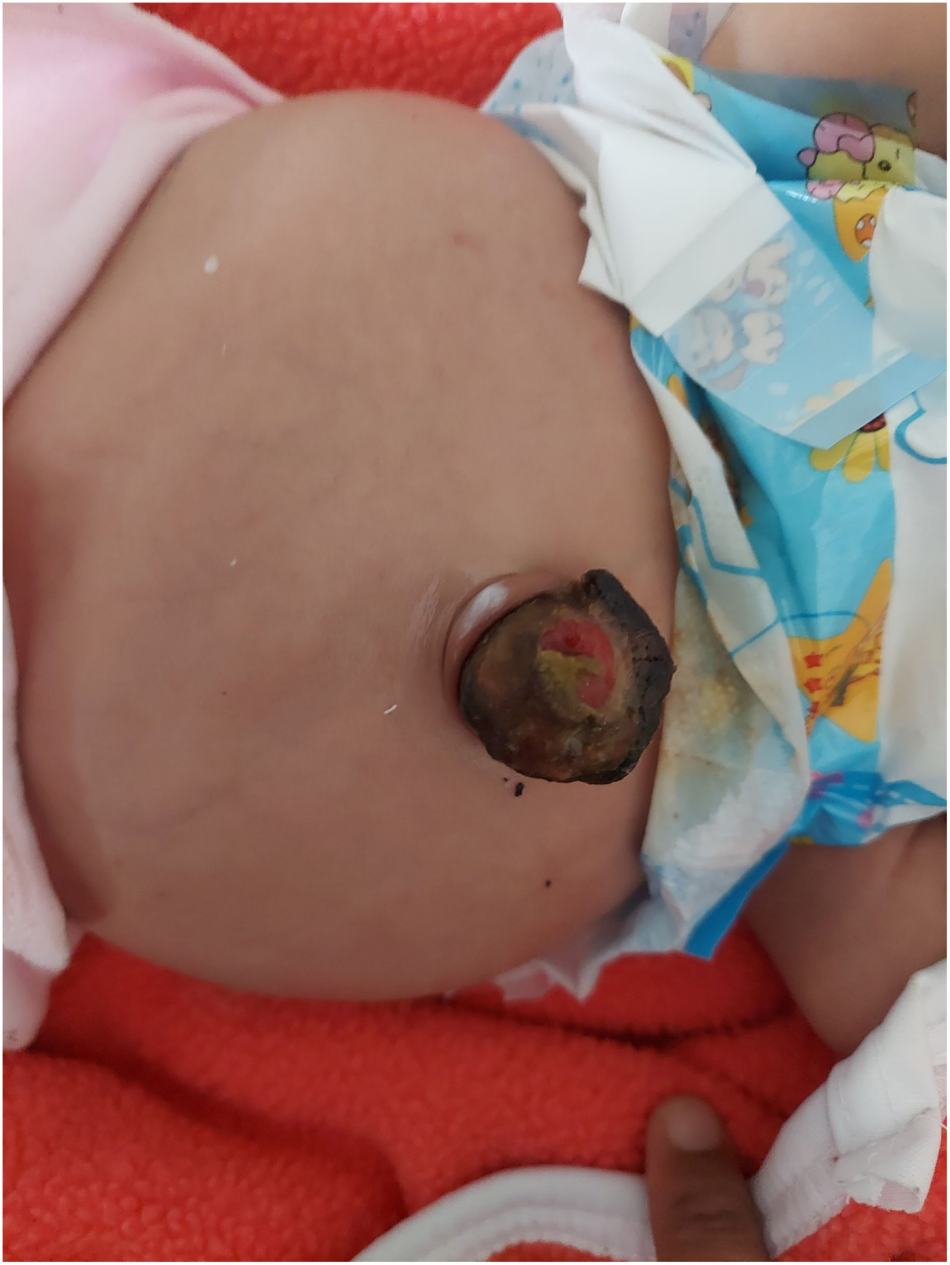
Protrusion of a mass via umbilical region at the time of presentation.

On examination, the child had a distended abdomen and a mass protruding through the umbilical region (Figure 1). He also had bilateral cryptorchidism. The rest of the findings including the vitals were within normal range. Similarly, he did not have any obvious facial deformities. Initial blood investigations revealed mild hyponatremia, mild hyperkalemia, and raised CRP level (Table 1). His blood culture isolated *Enterococcus species* and injectable antibiotics: Amikacin and Ampicillin were prescribed as per the sensitivity. The ultrasonography (USG) of his abdomen and pelvis showed that there was a congenital absence of his left kidney. The echocardiography of the baby revealed mild anterior tricuspid leaflet (ATL) prolapse with moderate tricuspid regurgitation (Gradient—22 mm Hg), presence of patent foramen ovale (PFO) with left to right shunt, and normal biventricular function (left ventricular ejection fraction [LVEF]: 69%).

**TABLE 1:** Laboratory parameters of the baby at the time of admission.

Parameters	Results	Normal reference used
Hematology		
Hemoglobin	173 gm/L	120‐180 gm/ L
Packed cell volume	48.8%	36%–54%
RBCs	4.66 million/mm^3^	4.5–5.5 million/mm^3^
Leucocytes	8600	4000–11,000
Platelets	204,000	150,000–450,000
Renal function test		
Creatinine	29 μmol/L	45–105 μmol/L
Urea	3.1 mmol/L	1.4–4.3 mmol/L
Na^+^	130 mEq/L	135–146 mEq/L
K^+^	5.4 mEq/L	3.5–5.2 mEq/L
Others		
CRP	11.61 mg//dL	0–6 mg/dL

Having the classical triad of prune belly syndrome and features of sepsis, the final diagnosis of prune belly syndrome with late‐onset neonatal sepsis was made. The omphalocele was repaired under general anesthesia with a circumferential periumbilical incision. During the procedure, an omphalocele containing a loop of viable bowel along with a Meckel's diverticulum (located 25 cm proximal to the ileocecal junction) was identified. The Meckel's diverticulum was excised through a wedge resection, the bowel was repaired, and umbiliculoplasty was done. Following the procedure, he was kept nil per oral (NPO) for 2 days and then breastfeeding was resumed.

## 4 CONCLUSIONS AND RESULTS

At the time of discharge, the child was playful, had stable vitals, tolerating breastfeeding well, and the wound was healthy. For cryptorchidism wait and watch policy was applied as both testes were in the superficial inguinal pouch. During the next 3‐month follow‐up, the child was playful, the incisional wound was healthy, and tolerating oral feeding well.

## 5 DISCUSSION

PBS, an *ACTG2* (actin gamma 2) visceral myopathy, is a highly morbid condition as the severe form is associated with stillbirth, renal failure, urinary infections, and pulmonary hypoplasia. There is an increased risk of early neonatal death mainly due to urosepsis and respiratory failure. This emphasizes the need for early diagnosis and management with a multidisciplinary approach for preventing poor prognosis.^7^, ^9^, ^10^, ^11^ The characteristic prune‐like wrinkled appearance of the abdominal wall is either due to the absence or deficiency of abdominal wall muscles.^7^ The baby in our case had a distended abdomen with an omphalocele due to a defect of abdominal wall musculature.

Most of the patients with PBS have unilateral or bilateral cryptorchidism. Cryptorchidism with or without an incompetent bladder neck (leading to retrograde ejaculation) and prostatic hypoplasia is associated with infertility in these patients. In addition, it is also associated with an increased risk of testicular malignancy.^12^ The physical examination of the neonate in the present case detected bilateral cryptorchidism. The urinary tract abnormalities in the patients with PBS range from mega bladder, dilated ureters, abnormal bladder neck, urethral stenosis, and hydronephrosis to dysplastic kidney with significant fibrosis, cystic kidney, and thinned‐out renal parenchyma.^11^ However, the baby being presented here had an absent left kidney. We suppose that this is the first‐ever reported case to have renal agenesis as a urinary tract abnormality in PBS.

During the prenatal period, PBS can be diagnosed by USG during the second trimester. When fetuses have a severe urogenital abnormality, oligohydramnios may be present. After birth, it is diagnosed by USG. To rule out vesicoureteral reflux (VUR) and to study the bladder outlet as well as the bladder emptying ability, voiding cystourethrogram (VCUG) is indicated. Other investigations are done to screen for associated organ system pathologies, for example, echocardiogram of the heart and ultrasound or CT scan of the abdomen for the cardiac and gastrointestinal anomalies, respectively.^1^, ^7^

The patient underwent abdominal USG that showed left renal agenesis. The parents were counseled for an abdominal CT scan of the child but it was refused. The echocardiography of the baby revealed moderate tricuspid regurgitation, the presence of PFO with left to right shunt, and normal biventricular function (LVEF: 69%). Renal function assessment is used to determine the prognosis of the disease and the lowest value of creatinine more than 0.7 mg/mL (61.89 μmol/L) is associated with poor prognosis. The child in the case had a normal renal function (Table 1).

Surgical intervention is the primary treatment for PBS, involving a series of operations such as urinary tract reconstruction, abdominoplasty, and orchidopexy.^13^ The timing and the sequence of these surgical techniques depend on individual cases based on the clinical severity and the types of defects. Urinary tract should be reconstructed early with priority in patients with recurrent febrile urinary tract infections (UTIs) or with progressive renal deterioration.^7^ Since, there was no abnormality in the renal tract, and the patient had a normal right renal system, no procedures were done involving the urinary system in our case. For cryptorchidism, orchidopexy is indicated. Since both the testicles of the baby were in the superficial inguinal pouch, we planned to go for the wait and watch approach for the descent. Similarly, for deficient or absent abdominal wall musculature, reconstructive procedures are a must. Depending on the condition of abdominal wall musculature, timing for abdominoplasty should be planned.^7^ The patient being presented here had a defect in the abdominal wall with an omphalocele, therefore, abdominoplasty was done as the first surgical procedure to prevent feeding difficulties, intestinal obstruction, and failure to thrive. In addition to surgical management, the medical management of PBS includes the treatment of associated complications and pathologies. For late‐onset neonatal sepsis, antibiotics and intravenous fluids were prescribed.

Therefore, it is critical for pediatrician to be aware of this rare entity in babies presenting with one or more obvious features of the triad as PBS can easily be missed. Since, the presence of a severe form of the disease is associated with bad outcomes, early diagnosis and immediate management of PBS is a must to prevent complications and mortality.

## AUTHOR CONTRIBUTIONS

**Siddinath Gyawali:** Conceptualization; data curation; formal analysis; investigation; methodology; resources; validation; writing – original draft; writing – review and editing. **Balkrishna Gyawali:** Conceptualization; data curation; writing – original draft; writing – review and editing. **Bhumika Ghimire:** Data curation; writing – original draft; writing – review and editing. **Bibek Shrestha:** Data curation; writing – original draft; writing – review and editing. **Pratima Khanal:** Data curation; writing – original draft; writing – review and editing. **Geha Raj Dahal:** Data curation; writing – original draft; writing – review and editing. **Dinesh Prasad Koirala:** Data curation; supervision; writing – original draft; writing – review and editing.

## FUNDING INFORMATION

The authors received no specific funding for this work.

## CONFLICT OF INTEREST STATEMENT

The authors declare that there is no conflict of interest regarding the publication of this paper.

## CONSENT

Written informed consent was obtained from the patient for publication of this case report and any accompanying images. A copy of the written consent will be made available for review to the editor‐in‐chief of this journal if asked. Written informed consent was obtained from the patient to publish this report in accordance with the journal's patient consent policy.

## Data Availability

Data will be made available on request.
